# Effects of bleaching-associated mass coral mortality on reef structural complexity across a gradient of local disturbance

**DOI:** 10.1038/s41598-018-37713-1

**Published:** 2019-02-21

**Authors:** Jennifer M. T. Magel, John H. R. Burns, Ruth D. Gates, Julia K. Baum

**Affiliations:** 1 Department of Biology, University of Victoria, Victoria, British Columbia V8P 5C2 Canada; 2 Hawai’i Institute of Marine Biology, University of Hawai’i, Kāne’ohe, Hilo, Hawai’i 96744 USA; 3 Marine Science Department, College of Natural and Health Sciences, University of Hawai’i at Hilo, Hilo, Hawai’i, 96720 USA

## Abstract

Structural complexity underpins the ecological functioning of coral reefs. However, rising ocean temperatures and associated coral bleaching threaten the structural integrity of these important ecosystems. Despite the increased frequency of coral bleaching events, few studies to date have examined changes in three-dimensional (3D) reef structural complexity following severe bleaching. The influence of local stressors on reef complexity also remains poorly understood. In the wake of the 2015-2016 El Niño-induced mass coral bleaching event, we quantified the effects of severe heat stress on 3D reef structural complexity across a gradient of local human disturbance. Using Structure-from-Motion photogrammetry we created 3D reconstructions of permanent reef plots and observed substantial declines in reef structural complexity, measured as surface rugosity and terrain ruggedness, and a detectable loss of habitat volume one year after the bleaching event. 3D reef complexity also declined with increasing levels of human disturbance, and with decreasing densities of branching and massive corals. These findings improve our understanding of the effects of local and global stressors on the structural foundation of coral reef ecosystems. In the face of accelerating climate change, mitigating local stressors may increase reef structural complexity, thereby heightening reef resilience to future bleaching events.

## Introduction

Habitat complexity has long been known to play an important role in structuring natural communities^1^. This is particularly true in highly-complex aquatic habitats such as coral reefs, given the unique physical challenges of the aquatic environment^2^. The physical structure provided by living corals, and the underlying topographic complexity of the substrate itself, play a critical role in the maintenance of biodiversity in coral reef ecosystems^3,4^. Complex structure supports high reef fish abundance and diversity^5–7^, mediates the effects of competition and predation on coral reefs^8^, facilitates the settlement of reef fish^9^, and may even make reefs more resilient to severe disturbances^10^. However, the increasing frequency and intensity of severe heat stress and associated coral bleaching events^11^ threatens the structural integrity of these vital ecosystems^12,13^.

Coral bleaching occurs when environmental stressors, such as elevated water temperatures^14,15^, disrupt the relationship between corals and their endosymbiotic algae (Symbiodiniaceae), resulting in expulsion of the algae from the coral tissue^16^ and heightening the chances of coral mortality^17^. In 2015-2016, heat stress associated with an extreme El Niño triggered the third major global coral bleaching event^18^, resulting in severe coral bleaching and mortality throughout all three tropical ocean basins^12^. Beyond impacts on live coral cover, extreme warming events such as this may also affect the three-dimensional (3D) structure of coral reef ecosystems. Mass coral bleaching can severely reduce reef carbonate budgets^19^, shifting reefs to a state of net erosion and limiting their ability to recover lost structure. This loss is compounded by increases in the abundance of common bioeroding organisms^20^ following mass bleaching, and the proliferation of dead coral substrate, which is more easily eroded^21^. Declines in reef structural complexity were reported following the 1998 mass coral bleaching event^3,22,23^, however these descriptions were primarily qualitative. To date, relatively few studies have quantified changes in 3D reef structural complexity following a severe heat stress event.

Quantitative measures of structural complexity on coral reefs have traditionally been made using the ‘chain-and-tape’ method for assessing linear rugosity^24^, however this technique is time-intensive, measures a small spatial area, and suffers from high variation due to the particular placement of the chain on the reef^25^. Recent advances in the quantification of coral reef structural complexity, most notably Structure-from-Motion (SfM) photogrammetry^26–28^, enable more precise and robust measures of fine-scale complexity, including a wider array of biologically-relevant structural complexity metrics^25,29^. However, the application of SfM photogrammetry to answering questions about reef structural change has so far been relatively limited (but see^30–34^). The few studies that have used SfM to quantify changes in reef complexity following coral bleaching have employed these techniques within only one^31,32^ to three^30^ reef plots, limiting our ability to understand the effects of bleaching on reef structure at a wider scale. Gaining a comprehensive understanding of the effects of bleaching-associated coral mortality on the physical structure of reefs and associated ecological processes will require quantification of these changes across environmental gradients and levels of human impact^35^ (e.g. from fishing^36^ or nutrient enrichment^37,38^, which can influence coral reef resilience and recovery from bleaching), using modern techniques capable of capturing fine-scale structural changes.

**Figure 1:**
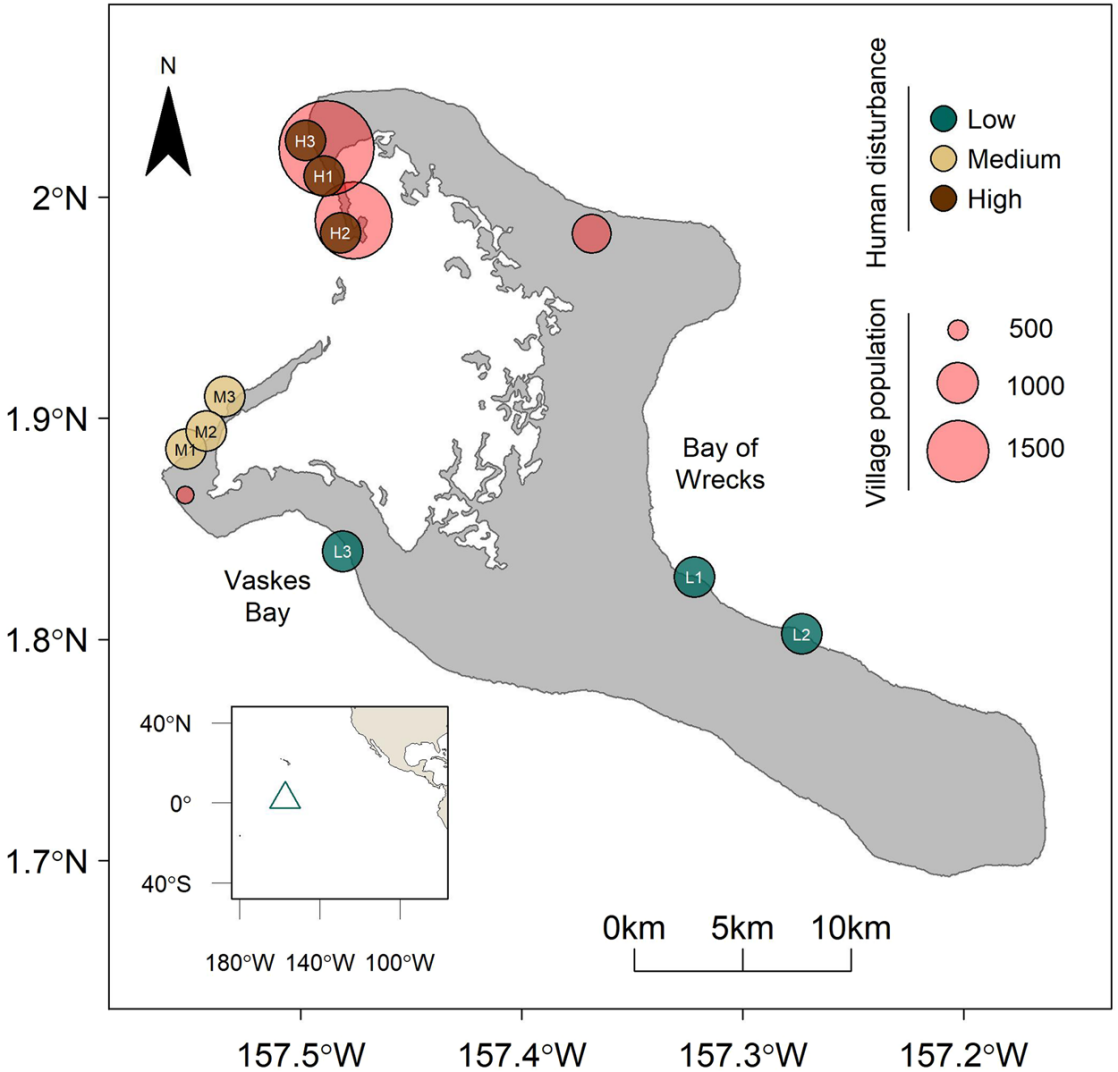
Map of forereef study sites and villages on Kiritimati, Republic of Kiribati. Sites are divided into three levels of local human disturbance, and villages (red circles) are scaled to human population size. Inset shows Kiritimati’s location in the equatorial central Pacific Ocean.

**Figure 2:**
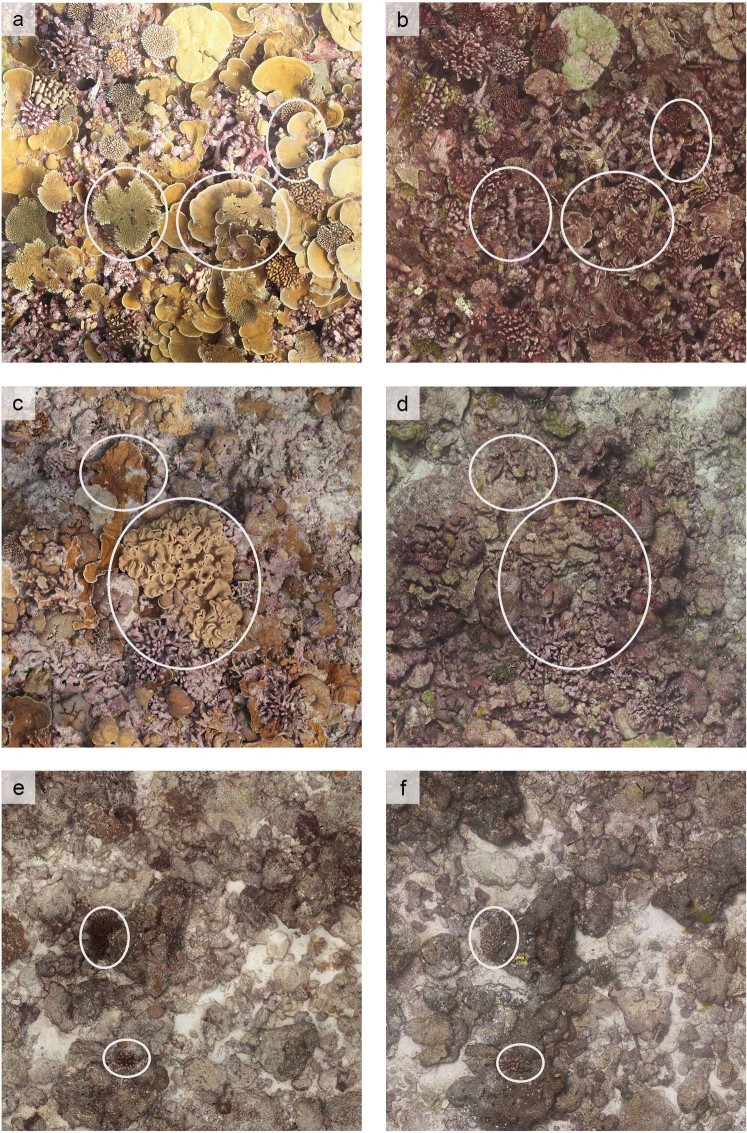
Photos of three permanent photoquadrats (PPQs) on Kiritimati, one from each of the low (**a**,**b**), medium (**c**,**d**), and high (**e**,**f**) human disturbance levels. Photos show the reef before (left) and after (right) the 2015-2016 El Niño and mass coral mortality event, and represent approximately 2 m × 2 m sections of each PPQ. In each row, the exact same area of the same PPQ is shown, with ellipses highlighting examples of visible changes in reef structure over time.

This study capitalized on a severe pulse heat stress event to examine the effects of bleaching-associated mass coral mortality on 3D reef structural complexity and the influence of underlying local human disturbance on these changes. During the 2015-2016 El Niño, coral reefs around Kiritimati (Republic of Kiribati) in the central equatorial Pacific Ocean were subjected to globally unprecedented levels of heat stress and suffered over 80% loss of coral cover by the end of the bleaching event (Baum, unpublished data). Kiritimati also presents the opportunity to examine the effects of local stressors on coral reef structural complexity: the atoll is characterized by a gradient of local human disturbance with the majority of its population concentrated on the northwest side of the island^39^ (Fig. 1), resulting in a diverse spectrum of reef states ranging from highly-degraded sites near the villages to near-pristine ones on the eastern side of the island (Fig. 2). Within the context of this ecosystem-scale natural experiment, we used photogrammetric techniques to quantify fine-scale reef structural complexity (surface rugosity, terrain ruggedness, curvature, and habitat volume) across the atoll’s disturbance gradient over the course of the bleaching event. Specifically, we aimed to (1) quantify the change in 3D reef structural complexity on Kiritimati resulting from the 2015-2016 El Niño, (2) determine the effect of local anthropogenic stressors on levels of structural complexity and the degree of structural change, and (3) examine the relationship between shifts in benthic composition and changes in coral reef structural complexity.

## Methods

### Study site and design

To examine changes in 3D reef structure following the 2015-2016 El Niño-induced mass coral bleaching event, we surveyed nine forereef sites around Kiritimati (Christmas Island, Republic of Kiribati) in the central equatorial Pacific Ocean (01°52′N 157°24′W, Fig. 1) in May 2015, before the onset of the El Niño, and again in July 2017, approximately 14 months after the end of the heat stress. Each site had previously been assigned to one of three human disturbance levels (low, medium, high) based on the intensity of fishing pressure^39^ and proximity to local villages (Baum *et al*., unpublished data). At each site we established three 4 m x 4 m permanent photoquadrats (PPQs) along the 10–12 m isobath to document temporal changes in coral reef benthic composition and structure. Stainless steel stakes were installed to demarcate each of the PPQ’s corners at all sites, and PPQs were spaced approximately 10 m apart from each other. We surveyed all three PPQs at each site in both time points, except at one low disturbance site (where inclement weather conditions only permitted sampling two PPQs in the first time point), for a total of 26 PPQs.

### Structural complexity

#### Photographic surveys and 3D model generation

We captured images of the reef substrate in each PPQ using SfM photogrammetry techniques for modelling coral reef habitats, described by Burns *et al*.^26^. Ground control points (GCPs) were placed at each corner of the plot at known depths and locations to enable accurate orthorectification of the resulting 3D models. A calibration grid and scale bar were also placed along the margin of the PPQ to validate the spatial accuracy of the 3D models. Photographs of the benthic substrate were collected while swimming in a boustrophodonic pattern approximately 3 m above the substrate. All images had 70–80% overlap and were taken from both planar and oblique angles. All photos were taken using a Nikon D750 digital SLR camera with a 24 mm lens (Nikon Canada Inc., Mississauga, Canada) and an Ikelite housing with an 8-inch hemispheric dome port (Ikelite Underwater Systems, Indianapolis, USA).

We used Agisoft PhotoScan software (Agisoft LLC., St. Petersburg, Russia) to process the images and produce spatially accurate 3D reconstructions and 2D orthophotomosaics of the surveyed PPQs. The 3D reconstruction workflow used photogrammetric techniques to produce a dense 3D point cloud, which represents the structure of the PPQ using data points in three-dimensional space, and a digital elevation model (DEM), which is a raster representing the 3D elevation of the reef substrate as a grid of squares. The orthophotomosaics are orthorectified, high-resolution images created using the source photos. The mosaics and DEMs are projected using the same local coordinate system so they can be layered to perform identification and measurement of individual coral colonies^26^. See Burns *et al*.^26^ for a detailed description of the model construction process.

#### Quantification of complexity metrics and habitat volumetric change

We quantified three structural complexity metrics using the 3D analyst and spatial analyst tools in ArcMap (ArcGIS 10.5, Environmental Systems Resource Institute, Redlands, USA). These geospatial metrics were specifically selected to capture variability in topographic complexity that is known to affect the biodiversity and abundance of marine organisms^26–33^. For each PPQ, the DEM was layered with the accompanying orthophoto and the area of the PPQ was digitized using the editor function in ArcMap. DEM cell size was set to 1.0 cm to capture fine-scale changes in the 3D physical structure of the reef. First, we quantified surface rugosity^28,40^ by using the ‘add surface information’ tool to quantify the ratio of 3D to 2D surface area for each PPQ. This metric is related to the traditional measure of linear reef rugosity but uses the ratio of 3D to 2D surface area rather than the ratio of contour to linear distance. Second, we used the ‘benthic terrain modeler’ toolbox to obtain a measure of terrain ruggedness for each PPQ, using a vector ruggedness measure. This metric quantifies surface complexity by measuring the three-dimensional dispersion of vectors orthogonal to the surface of the DEM. Vectors orthogonal to each grid cell are decomposed into their *x, y*, and *z* components, and a resultant vector is calculated within a 3 × 3 cell window centered on each cell. Larger values indicate areas of highly complex terrain. This metric simultaneously captures variation in both slope and aspect, and produces a measure of terrain ruggedness that is not strongly correlated with slope^41^. Third, we used the ‘curvature’ tool to quantify curvature values across the surface of each DEM. Curvature is measured by calculating the second derivative of the slope for each cell within a 3 × 3 window composed of the focal cell and its eight surrounding neighbours. This metric is a quantitative representation of convexities and concavities, which enables the identification of distinct topographic features such as peaks, ridges, channels, and planar regions^42,43^. Positive and negative curvature values represent upwardly concave or upwardly convex surfaces, respectively. Because both positive and negative values can correspond to high structural complexity, we used absolute values of curvature for our analysis.

To calculate volumetric change, we compared the 2015 and 2017 3D point clouds for each PPQ using the open-source software CloudCompare (v. 2.9.1, 2017). We aligned point clouds from the same PPQ by performing a rough alignment using the point pairs picking tool to select at least four matching pairs of points in each cloud, followed by a fine registration using the Iterative Closest Point (ICP) algorithm modified to allow for adjusted scaling of the source cloud to the reference cloud. Four permanent pins were installed at the corners of each 4 m x 4 m PPQ prior to this study. The pins from each PPQ were used as matching pairs to ensure the co-registration between point clouds was performed with invariant features that would not confound the temporal analysis. We then computed the volumetric change between the 2015 and 2017 point clouds using the 2.5D volume calculation tool, allowing us to quantify how much the volume of available reef habitat changed over time.

### Benthic composition

To quantify changes in benthic composition, we imported the two-dimensional orthophotomosaics of each PPQ into ArcMap for benthic analysis. For each PPQ, we identified all non-encrusting, intact hard coral colonies >5 cm in diameter to the lowest possible taxonomic level and assigned them a unique label so that the status of each colony could be tracked through time. In total, we enumerated 6655 individual coral colonies. To examine the relationship between the prevalence of different coral morphologies and changes in structural complexity, we also assigned each coral taxon to one of three coral growth forms (branching, plating, or massive; Supplementary Table S1) based on the Coral Traits Database (https://coraltraits.org/) and Veron^44^. For each PPQ, we calculated the density of each of these three coral growth forms (corals/m^2^) by dividing the abundance of each growth form by the area of the PPQ, to account for variation in PPQ size. We also measured the maximum diameter of each coral colony using the ‘measure’ tool and used these measurements to calculate ‘coral cover’ for each growth form in each PPQ. However, we found that coral abundance, coral density, and coral cover were highly correlated (*r* = 0.83–0.99) for each of the three growth forms, so we proceeded with a single metric (coral density) for statistical modelling.

### Statistical analyses

We conducted all statistical analyses using the statistical software R version 3.3.1^45^. For our analysis, we used model selection and multi-model inference^46^ to examine the influence of local and global stressors on coral reef surface rugosity, terrain ruggedness, and absolute curvature. For each structural complexity metric, we fit linear mixed-effects models with heat stress, local human disturbance, and the density of each coral growth form as fixed effects, and site as a random effect (to account for non-independence amongst PPQs at the same site). Heat stress was modelled as a categorical variable with two levels (‘before’, ‘after’), because *in situ* temperature loggers (SBE-56, Sea-Bird Electronics, Bellevue, USA) deployed at sites around Kiritimati indicated a consistent change in water temperature around the atoll throughout the heat stress event (Supplementary Fig. S1). Local human disturbance was also modelled as a categorical variable with three levels (‘low’, ‘medium’, ‘high’), while the densities of each coral growth form were modelled as continuous variables. Prior to analysis, all continuous input variables were standardized to a mean of zero and a standard deviation of 0.5 using the ‘rescale’ function in the *arm* package^47^, to allow for direct comparison of the effect sizes of different variable types^48^. We also explored the possibility of including wave energy as a fixed effect, however this was ultimately deemed to be unnecessary because of minimal variability across the sampled sites (see Supplementary Methods).

To account for unequal variance in the residuals of our full model, we also employed residual variance structures. Variance structures provide a means of modelling identifiable structure in the residuals without the penalty of adding additional parameters to the model^49^. We used AIC corrected for small sample sizes (AIC_c_)^50^ to compare models with and without residual variance structures, and identify metrics for which inclusion of a variance structure improved model fit. For the surface rugosity and absolute curvature models, we applied a varIdent residual variance structure to account for unequal variance between time points (before vs. after) in the full model. The same variance structure was tested for terrain ruggedness but did not improve model fit.

For each structural complexity metric, we evaluated 40 models by fitting every combination of variables, including a two-way interaction between heat stress and local human disturbance. To perform model selection, we used AIC_c_ to compare models and produce a top model set comprised of all models within 4 ΔAIC_c_ of the best model. Within this top model set, we calculated multi-model-averaged parameter estimates and 95% confidence intervals for each predictor variable. We also determined the relative variable importance (RVI) for each predictor retained in the top model set, calculated as the sum of Akaike weights across all models containing that variable. RVI is a metric that allows for the ranking of predictor variables, with the most important variable having a maximum possible value of 1.0^46^. Assumptions of normality and homogeneity of variance were evaluated graphically for all models retained in the top model set, and in each case appeared to be reasonable fits. Models were fit using the R package *nlme*^51^, and model selection was performed using the package *MuMIn*^52^.

## Results

Ground sampling distance (GSD) is the distance between two consecutive pixel centers and serves as a standard measure of spatial resolution in remote sensing. Smaller GSD values correspond to greater spatial resolution and ability to detect intricate spatial features. The GSD (resolution/pixel) of all 3D reconstructions was less than 0.01 m/pix. All DEMs were rendered with a cell size of 1.0 cm, which is within the range of the GSD computed for all models. DEM cell size was set to 1.0 cm as this resolution has been shown to be effective at capturing temporal changes in coral reef habitat complexity^30–33^. The structural complexity metrics used in this study are quantified in 3 × 3 cell windows of the DEM, thus our approach detected changes in topographic complexity occurring at a 3.0 cm resolution.

### Structural complexity metrics

Our models indicated that reef structural complexity was determined by a combination of local human disturbance, heat stress, and the densities of branching and massive corals. However, the strongest predictors varied between surface rugosity, terrain ruggedness, and curvature, and no predictor consistently explained 3D reef structural complexity across all three metrics. While the interaction between heat stress and local human disturbance appeared in the top model sets for both surface rugosity and terrain ruggedness, the effect of this predictor was fairly weak (maximum relative variable importance of 0.43; Table 1), suggesting that local stressors do not strongly influence patterns of structural change on coral reefs following a heat stress event.

**Table 1:** Top models describing three distinct components of coral reef habitat complexity: surface rugosity (a), terrain ruggedness (b), and absolute curvature (c). Plus signs indicate the presence of variables within each model in the top model set.

Rank	Heat	Dist.	Branching	Plating	Massive	Heat*Dist	*df*	AICc	ΔAICc	*w*	*R* ^2^
(a) Surface rugosity											
1	+	+			+		8	14.89	0.00	0.17	0.54
2	+	+	+		+		9	15.11	0.22	0.16	0.59
3	+	+			+	+	10	16.05	1.16	0.10	0.63
4	+	+					7	16.13	1.24	0.09	0.50
5	+	+	+				8	16.27	1.38	0.09	0.55
6	+	+	+		+	+	11	16.82	1.93	0.07	0.65
7		+	+		+		8	17.16	2.27	0.06	0.57
8	+	+		+	+		9	17.32	2.42	0.05	0.56
9	+	+	+	+	+		10	18.19	3.29	0.03	0.59
10	+	+		+			8	18.66	3.76	0.03	0.51
RVI	**0.92**	**0.94**	0.49	0.20	0.72	0.22					
(b) Terrain ruggedness											
1	+	+	+		+		8	−304.36	0.00	0.32	0.80
2	+	+	+		+	+	10	−303.18	1.18	0.18	0.83
3	+	+	+	+	+		9	−303.11	1.24	0.17	0.80
4	+	+	+	+	+	+	11	−301.32	3.04	0.07	0.83
5	+	+	+				7	−301.08	3.27	0.06	0.76
6		+	+	+	+		8	−300.69	3.67	0.05	0.77
RVI	**0.92**	**0.96**	**0.95**	0.36	**0.90**	0.28					
(c) Absolute curvature											
1					+		5	397.40	0.00	0.27	0.23
2			+		+		6	398.94	1.54	0.13	0.27
3				+	+		6	399.34	1.94	0.10	0.25
4	+				+		6	399.73	2.32	0.09	0.22
5		+					6	400.32	2.92	0.06	0.26
6		+			+		7	400.55	3.14	0.06	0.28
7	+		+		+		7	400.66	3.25	0.05	0.25
8	+			+	+		7	401.39	3.99	0.04	0.24
RVI	0.25	0.25	0.30	0.24	**0.84**	0.00					

Heat = heat stress; Dist. = local human disturbance; Branching = branching coral density; Plating = plating coral density; Massive = massive coral density; *df* = degrees of freedom; AIC_c_ = AIC corrected for small sample sizes; ΔAIC_c_ = difference from the lowest AIC_c_ value, all models within ΔAIC_c_ of 4 are shown; *w* = model weight; *R*^2^ = conditional *R*^2^ (proportion of variance explained by both fixed and random effects); RVI = relative variable importance.

#### Surface rugosity

**Figure 3:**
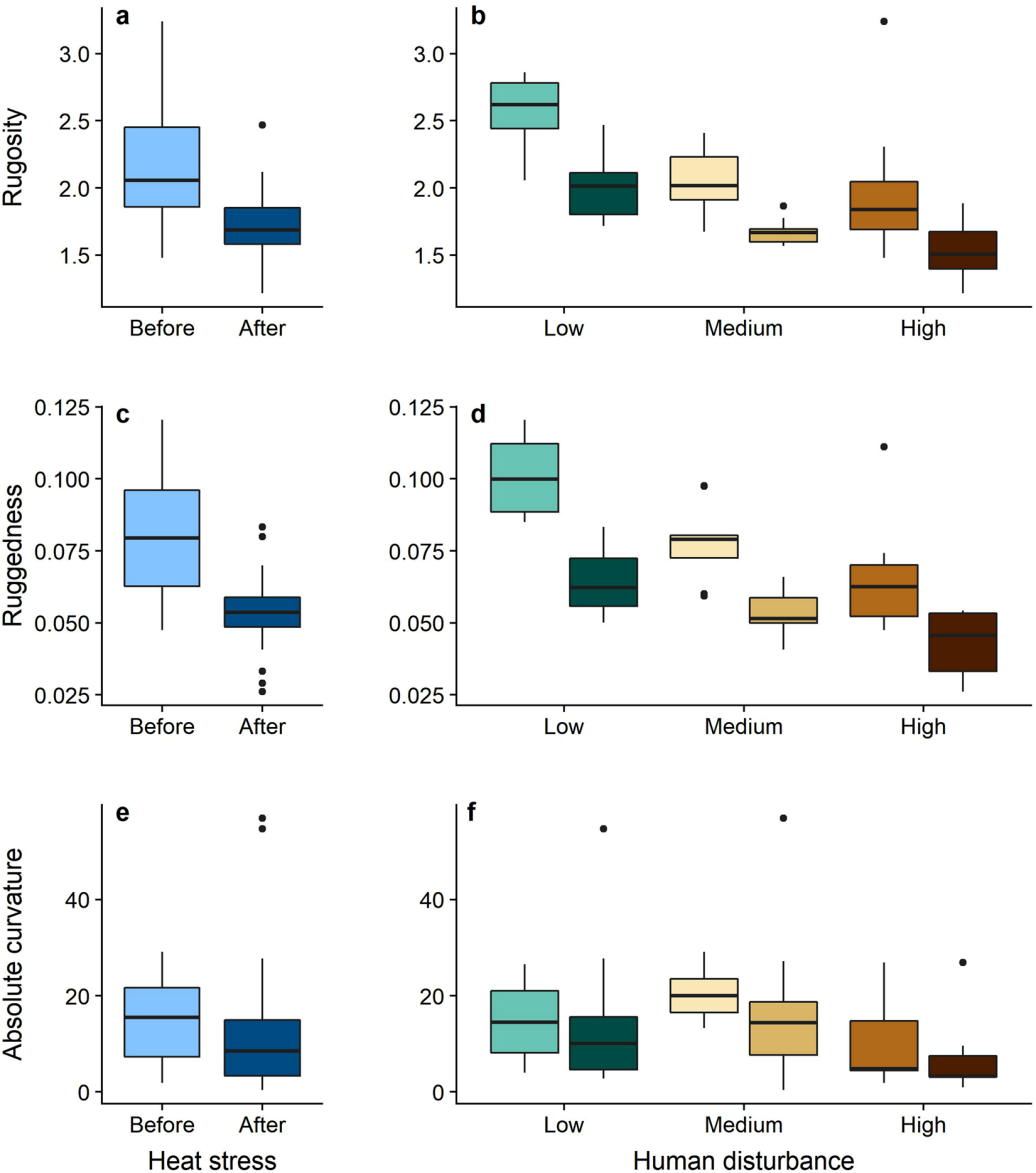
Levels of coral reef surface rugosity (**a**,**b**), terrain ruggedness (**c**,**d**), and absolute curvature (**e**,**f**) before and after the 2015-2016 heat stress event. Plots represent changes in each metric on the atoll overall (left) and across the human disturbance gradient (right). For each disturbance level, lighter boxes indicate 2015 values and darker boxes indicate 2017 values.

**Figure 4:**
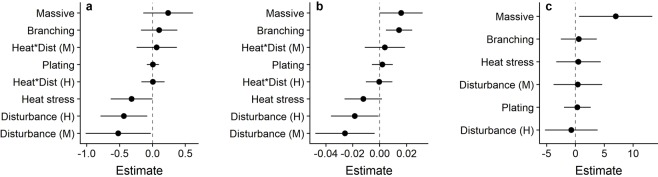
Multi-model-averaged parameter estimates and 95% confidence intervals for surface rugosity (**a**), terrain ruggedness (**b**), and absolute curvature (**c**). Heat*Dist refers to the interaction between heat stress and local human disturbance (M = medium disturbance, H = high disturbance).

Reef surface rugosity was heavily influenced by both local human disturbance and heat stress. Disturbance was the strongest predictor, with a relative variable importance of 0.94 across all models (Table 1). Surface rugosity was highest at sites with low local human disturbance and declined as disturbance increased (Figs 3b and 4a). Rugosity was also negatively impacted by heat stress (Fig. 4a). All 26 PPQs experienced declines in surface rugosity following the mass coral bleaching and mortality event, with mean values of rugosity across all sites declining from 2.189 ± 0.452 to 1.730 ± 0.268 (mean ± standard deviation [SD], Fig. 3a). Massive and branching corals positively influenced reef rugosity, although morphologies had less influence than either disturbance or heat stress (Fig. 4a; Table 1).

#### Terrain ruggedness

Terrain ruggedness was predicted by local human disturbance, the densities of both branching and massive corals, and heat stress, all of which had a relative variable importance ≥0.90 (Table 1). Like surface rugosity, ruggedness was highest at low disturbance sites and declined as disturbance increased (Fig. 3d). Ruggedness was also positively related to the density of branching and massive corals (Fig. 4b). Although slightly weaker than the effect of local disturbance, heat stress still had a negative effect on terrain ruggedness (Fig. 4b), and as with rugosity, all 26 PPQs showed declines in terrain ruggedness one year after the mass coral bleaching event from a mean value of 0.081 ± 0.021 in 2015 to 0.053 ± 0.013 in 2017 (Fig. 3c).

#### Absolute curvature

Massive coral density was the only important predictor of absolute curvature, having a relative variable importance of 0.84 across all models (Table 1). Curvature was higher in PPQs with greater densities of massive corals (Fig. 4c). Unlike terrain ruggedness and surface rugosity, which peaked under low levels of disturbance, curvature appeared to be slightly higher at intermediate levels of human disturbance (Fig. 3f). Following the heat stress event, only 15 out of 26 individual PPQs suffered declines, with the remaining 11 PPQs experiencing increases in curvature. As such, absolute curvature values remained relatively stable (15.07 ± 8.35 prior to the heat stress event vs. 13.32 ± 14.83 afterward; Fig. 3e).

### Habitat volume

**Figure 5:**
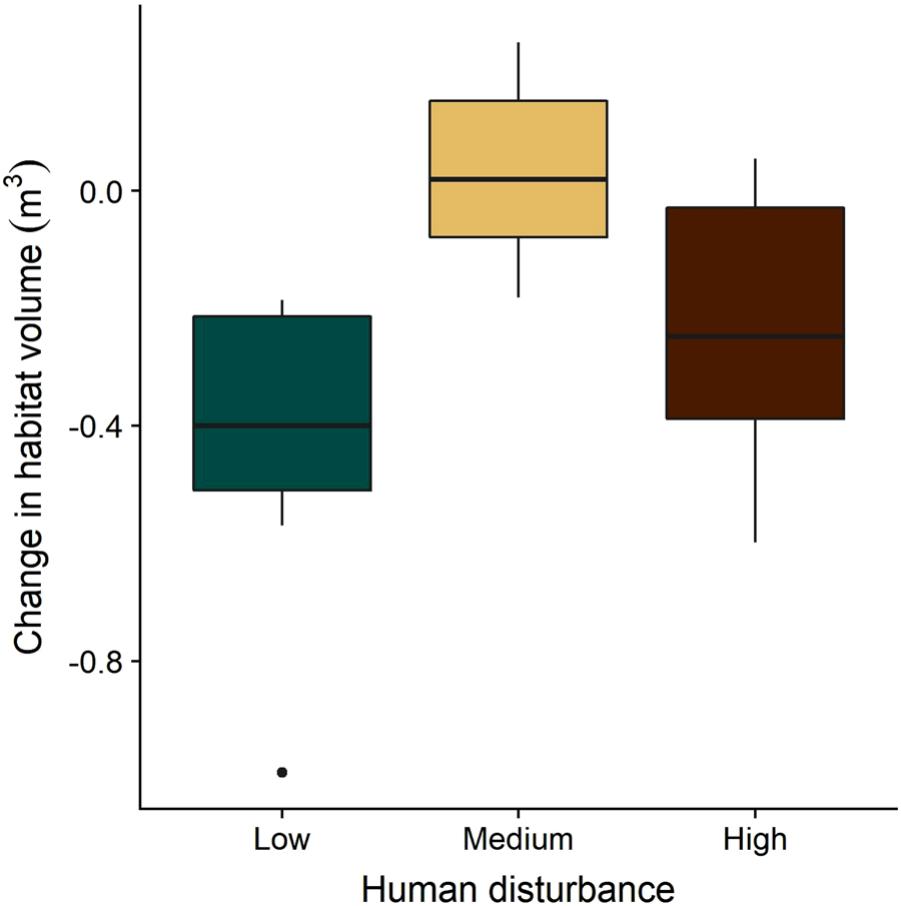
Net change in habitat volume within individual 4 m × 4 m PPQs across the local human disturbance gradient. Changes occurred over an approximately two-year period (May 2015–July 2017), during and after the 2015-2016 heat stress event.

**Figure 6:**
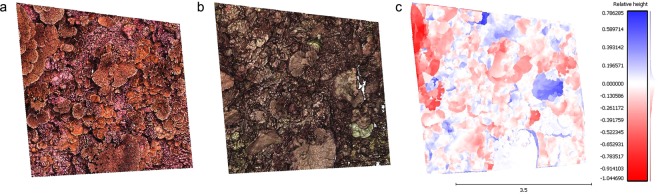
Comparison of 3D point clouds from before (**a**) and after (**b**) the 2015-2016 El Niño for the PPQ experiencing the greatest decline in habitat volume. The CloudCompare 2.5D volume calculation tool calculates the difference in height (m) between each point in the ‘before’ and ‘after’ point clouds, and depicts the loss (red) and gain (blue) of reef substrate over time (**c**).

Habitat volume declined in 19 out of 26 PPQs following the heat stress event, with sites at low and high levels of local human disturbance suffering a greater loss in habitat volume than those at medium disturbance (Fig. 5). The PPQ experiencing the greatest decline in habitat volume (a net loss of 0.990 m^3^ of reef substrate) was located at one of the low disturbance sites (Fig. 6). All PPQs exhibited areas of both loss (red) and gain (blue) of habitat volume through time. However, on average, individual PPQs suffered a net loss in habitat volume of 0.204 ± 0.283 m^3^ of substrate within one year of the severe heat stress event.

## Discussion

Given the importance of 3D structural complexity in mediating the responses of reefs to disturbances such as coral bleaching^10^, understanding the drivers of reef complexity will be critical for the preservation of reefs under future scenarios of climate change. Here, we examined three different metrics of coral reef structural complexity (surface rugosity, terrain ruggedness, and absolute curvature) and found that declines in both rugosity and terrain ruggedness were detectable only a year after the 2015-2016 El Niño and mass coral bleaching event, with some PPQs losing close to 1 m^3^ of reef substrate (Figs 3–5). The declines in surface rugosity that we observed corroborate the findings of Burns *et al*.^31^ and Couch *et al*.^32^, who used SfM techniques to document changes in reef complexity on a smaller number of plots within one year of severe bleaching events in the Hawaiian Islands. Conversely, our results suggest that curvature was not substantially affected by the heat stress event, which is also consistent with the findings of Burns *et al*.^31^. Additionally, we provide what is, to the best of our knowledge, the first evidence that terrain ruggedness is negatively impacted by heat stress in coral reef ecosystems. Although we did not find strong evidence for an interaction between heat stress and local human disturbance, we did observe a decrease in variance around the mean values of surface rugosity and terrain ruggedness from before to after the heat stress event. This suggests that the declines in reef structure measured here are leading to a homogenization of structural complexity values, with sites across the human disturbance gradient becoming increasingly similar in their levels of structural complexity.

Despite the influence of baseline levels of structural complexity on reef resilience and recovery capacity^10^, few studies to date have examined the effects of local anthropogenic stressors on 3D reef structure. Our results suggest that 3D reef complexity declines with increasing levels of local human disturbance. Reefs exposed to intermediate to high levels of local disturbance due to stressors such as fishing, pollution, and coastal infrastructure had substantially lower levels of structural complexity compared to reefs with very little local human disturbance. However, because our human disturbance metric is based on a combination of fishing pressure and proximity to local villages (which may serve as a proxy for stressors such as pollution and sedimentation), we are unable to pinpoint the precise mechanism behind this pattern. Possible drivers include high levels of sedimentation associated with coastal infrastructure that limit coral recruitment^53^, overfishing of important functional groups such as herbivores that help to maintain the reef in a coral-dominated state^54^, and direct physical damage to coral structure by fishing gear^55^ or boat anchors. However, more research will be needed in the future to tease apart and test the impacts of each of these factors on reef structural complexity.

The abundance of particular coral growth forms (e.g. complex branching corals) is also thought to be an important factor driving levels of 3D structural complexity on coral reefs. Here, we found evidence that levels of reef structural complexity are heavily influenced by the densities of both branching and massive corals (Fig. 4, Table 1). While some previous studies have found negative associations between the abundance of branching corals and structural complexity^56^, we found that branching coral density had a positive impact on all three structural complexity metrics, with this effect being particularly strong for terrain ruggedness. Massive coral density was also positively related to all three metrics, with the strongest effects for terrain ruggedness and curvature. This strong influence of massive coral density on reef structural complexity may seem counterintuitive, given the domed structure of many massive coral genera. However, the massive coral assemblage on Kiritimati includes corals with a variety of finer-scale morphologies that may contribute to increased structural complexity. For example, certain submassive coral species such as *Favites pentagona* and *Goniastrea stelligera* can take on columnar forms, while *Pavona duerdeni* may produce ridge-like structures. The contribution of massive corals to measures of reef complexity may also depend both on the scale at which complexity is measured and the particular metrics that are quantified. For example, massive corals, with their rounded surfaces, may achieve higher values for metrics based on vector dispersion (e.g. terrain ruggedness) or changes in slope (e.g. curvature) compared to plating corals, which have large planar surfaces. These predictions reflect the findings of previous research on reef structure suggesting that complexity is higher for larger massive and branching coral colonies than for plating colonies^40,57,58^.

The lack of a relationship between plating coral density and reef structural complexity is nevertheless surprising, given the recognized role of tabular corals as “keystone structures” on coral reefs^59^ and the high surface-area-to-volume ratio of these species, which would be expected to contribute to high levels of surface rugosity. However, our results may be an artifact of the type of 3D model used in our analysis. While digital elevation models (DEMs) are commonly used in photogrammetry to represent 3D surfaces, including in other coral reef studies^26,31,32,57^, DEMs are projected from one planar angle and thus are not truly three-dimensional, since they cannot model multiple *z* points at a single (*x,y*) coordinate^60^. Given that the structural function of plating corals relies largely on the existence of sheltered spaces beneath the coral plates^61^, analyzing reef structure from 2.5D DEM projections may underestimate the complexity of foliose and tabulate coral morphologies, compared to other coral growth forms^40^. These effects may be especially severe in areas where plating corals form complex, multi-tiered structures. As such, we suggest that future studies work towards developing methods to extract complexity data from true 3D digital surface models.

The strong dependence of 3D reef structural complexity on the presence of branching corals has implications for the maintenance of reef structure under future climate change. Branching corals, such as acroporids and pocilloporids, are highly susceptible to heat stress and bleaching-associated mortality^62^ as well as physical damage from storms^63^. Based on our PPQ benthic data, the density of live branching corals on Kiritimati declined by 95% following the mass coral bleaching event. Although many dead branching coral skeletons remain and continue to provide structure to the reef, it is likely that these structures will soon erode, resulting in further declines in reef complexity. While our results, and those of previous studies^64^, suggest that massive corals may play an important structural role on coral reefs, it remains to be seen how the increased dominance of this coral growth form will affect the ecological functioning of the reefs around Kiritimati. Shifts in coral assemblages from complex reef-building species to smaller, weedy coral species have previously been shown to result in substantial declines in coral reef calcification rates and linear rugosity values^65^. Continued monitoring of the reef system will be necessary to determine the magnitude of the shift in benthic composition following the mass coral mortality event, and how this change impacts both the physical structure and ecological function of the reef over the long term.

Given the many important ecological processes and ecosystem services facilitated by coral reef structural complexity, the loss of reef structure has negative implications for both marine organisms and human coastal communities. Declines in reef complexity are expected to compromise coral reef fisheries, potentially leading to up to a three-fold decline in fisheries productivity in severe cases^66^. This problem is of particular concern for local communities on small, isolated islands such as Kiritimati, where the majority of people depend on reef fisheries for income and subsistence and opportunities for alternative livelihoods are scarce^39,67^. A key question for future research will be to determine how levels of structural decline similar to those observed here, as well as those occurring in subsequent years with further degradation of the reef substrate, impact the structure of reef fish assemblages and populations of other ecologically and socioeconomically-important reef organisms.

Prior to the advent of SfM photogrammetry techniques, studies documenting bleaching-induced changes in reef complexity using conventional methods usually took place several years after the end of a severe disturbance event, and documented the extreme and highly-visible collapse of reef structure^3,22,23^. While it is vital to understand the effects of severe levels of reef degradation on coral reef communities, studying multiple time points along the trajectory of reef degradation will allow us to gain a better understanding of the rates and impacts of fine-scale structural change, and detect threshold levels of structural complexity below which the ecological functioning of the reef is impaired. As such, it is vital that coral reef monitoring programs incorporate modern methods for quantifying structural complexity into their standard reef monitoring protocols. Photogrammetry techniques provide a time- and cost-effective approach that can be used to this end to objectively quantify multiple measures of fine-scale structural complexity on coral reefs.

Coral reefs are currently being impacted at multiple scales by a suite of natural and anthropogenic disturbances. In this study we have shown that levels of 3D structural complexity, a vital component of healthy coral reef ecosystems, are impacted by both local and global stressors. Our finding that local human disturbance is a strong predictor of structural complexity suggests that chronic local stressors may have indirect impacts on reef recovery potential through their influence on reef structure. As such, management should focus on mitigating local stressors in order to maintain reefs at ecologically functional levels of structural complexity. However, action at the local level will also need to be accompanied by policy changes at the global scale. Recent research suggests that limiting global warming to 2 °C, the upper limit of the recent Paris Agreement, will still result in annual severe bleaching on the majority of coral reefs within the next few decades^68^. Our results provide further evidence for the negative effects of ocean warming on coral reef ecosystems, demonstrating the need for drastic reductions in greenhouse gas emissions and support for the development of sustainable low-carbon infrastructure.

## Supplementary information

Supplementary_Materials

## Data Availability

The data and R code used in the statistical analysis for this study are publicly available through Zenodo (10.5281/zenodo.1615675).
