# Supplementary material for: Effects of bleaching-associated mass coral mortality on reef structural complexity across a gradient of local disturbance
Source: Sci Rep. 2019 Feb 21;9:2512. doi: 10.1038/s41598-018-37713-1 (PMC6385266; doi:10.1038/s41598-018-37713-1)
Supplement: Supplementary file 1 — Supplementary_Materials [file 41598_2018_37713_MOESM1_ESM.pdf]

Supplementary information

Effects of bleaching-associated mass coral mortality on reef structural complexity across a gradient of local disturbance

Jennifer M.T. Magel[1*], John H.R. Burns[2,3], Ruth D. Gates[2], Julia K. Baum[1,2]

[1] Department of Biology, University of Victoria, Victoria, British Columbia, V8P 5C2, Canada
[2] Hawai‘i Institute of Marine Biology, University of Hawai‘i, Kāne‘ohe, Hawai‘i, 96744, USA
[3] Marine Science Department, College of Natural and Health Sciences, University of Hawai‘i at Hilo, Hilo, Hawai‘i, 96720, USA

*Corresponding author
E-mail: jenn.magel@gmail.com (JMTM)

Supplementary methods

Wave energy

The influence of physical factors such as wave energy may also impact benthic community composition and structure, and this relationship has been well-documented in coral reef ecosystems[1–3]. As such, we also explored the possibility of incorporating wave energy in our structural complexity models. Firstly, to determine whether there was any spatial autocorrelation remaining in our models that was not accounted for by the local human disturbance variable, we plotted variograms for the full models of each structural complexity metric. Given our relatively small sample sizes, the resulting variograms exhibited considerable residual variation without any clear patterns. However, visual assessment of variance suggested that spatially clustered sites did not exhibit increased residual correlation, as they might be expected to if there was an important covariate missing from the model[4]. Furthermore, adding wave energy to the models did not appear to improve measures of variance. This suggests that the influence of spatial autocorrelation amongst our sites was already largely accounted for by the variables present in the model. While we suspect that this was mainly due to inclusion of the local human disturbance variable, some of the spatial autocorrelation may also have been accounted for by the inclusion of coral growth forms in our model, given the relationships between potentially-confounding biophysical factors such as wave energy and benthic structure[1].

In addition, remotely-sensed wave energy data from the Marine Socio-Environmental Covariates data set (shiny.sesync.org/apps/msec/)[5] suggests that Kiritimati experiences remarkably little variation in wave energy, with long-term mean wave energy values at our study sites only ranging from ~25-27 kW m^{-1}. In comparison, studies conducted at other locations in the Northern Line Islands, at sites comparable in depth to those surveyed here, report intra-island

variations in wave energy of at least 15 kW m^{-1} (e.g. long-term mean wave energy of ~15-30 kW m^{-1} at Kingman Reef)[5]. On Kingman, areas of higher wave energy are associated with lower hard coral cover, as well as lower cover of the genus *Acropora*[5]. However, the sites on Kiritimati with the highest coral cover and structural complexity (much of which is provided by *Acropora*) are also those with the highest mean wave energy, which contradicts the expected effects of wave energy on benthic structure. This suggests that even if the observed gradient in wave energy across our sites was sufficient to drive differences in reef structural complexity, the benthic community on Kiritimati has likely become decoupled from these natural biophysical drivers due to the presence of chronic local human disturbance[6]. Therefore, given our assessment of spatial autocorrelation in the models, the low variability in wave energy on Kiritimati, and the potential biophysical decoupling, we feel that adding wave energy to our models would not improve the analysis or interpretation of the data.

References

1. Bradbury, R. H. & Young, P. C. The effects of a major forcing function, wave energy, on a coral reef ecosystem. *Mar. Ecol. Prog. Ser.* **5**, 229–241 (1981).
2. Dollar, S. J. Wave stress and coral community structure in Hawaii. *Coral Reefs* **1**, 71–81 (1982).
3. Williams, G. J. *et al.* Benthic communities at two remote Pacific coral reefs: effects of reef habitat, depth, and wave energy gradients on spatial patterns. *PeerJ* **1**, e81; 10.7717/peerj.81 (2013).
4. Zuur, A. F., Ieno, E. N., Walker, N. J., Saveliev, A. A. & Smith, G. M. *Mixed Effects Models and Extensions in Ecology with R* (Springer-Verlag, 2009).
5. Yeager, L. A., Marchand, P., Gill, D. A., Baum, J. K. & McPherson, J. M. Marine Socio-Environmental Covariates: queryable global layers of environmental and anthropogenic variables for marine ecosystem studies. *Ecology* **98**, 1976 (2017).
6. Williams, G. J., Gove, J. M., Eynaud, Y., Zgliczynski, B. J. & Sandin, S. A. Local human impacts decouple natural biophysical relationships on Pacific coral reefs. *Ecography* **38**, 751–761 (2015).

Table S1. Coral species (or species groups) identified from orthophotomosaics of permanent photoquadrats (PPQs), classified by growth form (branching, massive, or plating) and Family**.** Coral taxonomy and name synonymy retrieved from WoRMS (http://www.marinespecies.org/). Growth forms were assigned based on the Coral Traits Database (https://coraltraits.org) and Veron (2000).

Family	Species	Notes
**Branching corals**		
Acroporidae	*Acropora* spp. (corymbose morphology)	Includes *A. rosaria* (synonym: *A. loripes*), *A. subulata*, and hybrids of these species
Acroporidae	*Acropora gemmifera*	May include *A. globiceps*
Pocilloporidae	*Pocillopora grandis*	Synonym: *Pocillopora eydouxi*
Pocilloporidae	*Pocillopora meandrina*	
Pocilloporidae	*Pocillopora* spp.	Any *Pocillopora* that could not be classified to species due to a lack of defining morphological characteristics (e.g. recruits)
**Massive corals**		
Acroporidae	*Astreopora* spp.*	Includes *A. cucullata*, *A. myriophthalma*, and *A. suggesta*
Agariciidae	*Pavona duerdeni*	
Fungiidae	*Danafungia* spp., *Lithophyllon* spp., *Lobactis* spp., *Pleuractis* spp. (synonym: *Fungia* spp.)	Includes *Danafungia scruposa* (synonym: *F. corona*), *D. horrida* (synonym: *F. danai*), *Lithophyllon concinna* (synonym: *F. concinna*), *Lobactis scutaria* (synonym: *F. scutaria*), and *Pleuractis granulosa* (synonym: *F. granulosa*)
Fungiidae	*Herpolitha limax*	
Fungiidae	*Sandalolitha robusta*	
Lobophylliidae	*Lobophyllia hemprichii**	
Merulinidae	*Astrea* spp. (synonym: *Montastrea* spp.)*	May include *A. annuligera* (synonym: *M. annuligera*) and *A. curta* (synonym: *M. curta*)
Merulinidae	*Dipsastraea matthaii*	Synonym: *Favia matthaii*
Merulinidae	*Dipsastraea speciosa*	Synonym: *Favia speciosa*
Merulinidae	*Favites halicora*	
Merulinidae	*Favites pentagona**	
Merulinidae	*Goniastrea stelligera**	Synonym: *Favia stelligera*
Merulinidae	*Hydnophora microconos*	
Merulinidae	*Platygyra* spp.	Primarily *P. daedalea*; may include *P. contorta*, *P. ryukyuensis*, and *P. sinensis*
Poritidae	*Porites lobata*	May include *P. evermanni* and *P. lutea*
Psammocoridae	*Psammocora haimiana*	
**Plating corals**		
Acroporidae	*Acropora hyacinthus*	
Acroporidae	*Montipora aequituberculata* (foliose morphology)	
Dendrophylliidae	*Turbinaria reniformis*	
Lobophylliidae	*Echinophyllia aspera**	
Merulinidae	*Hydnophora exesa**	

*Coral taxa documented as having multiple growth forms were assigned to the growth form most commonly observed on Kiritimati.

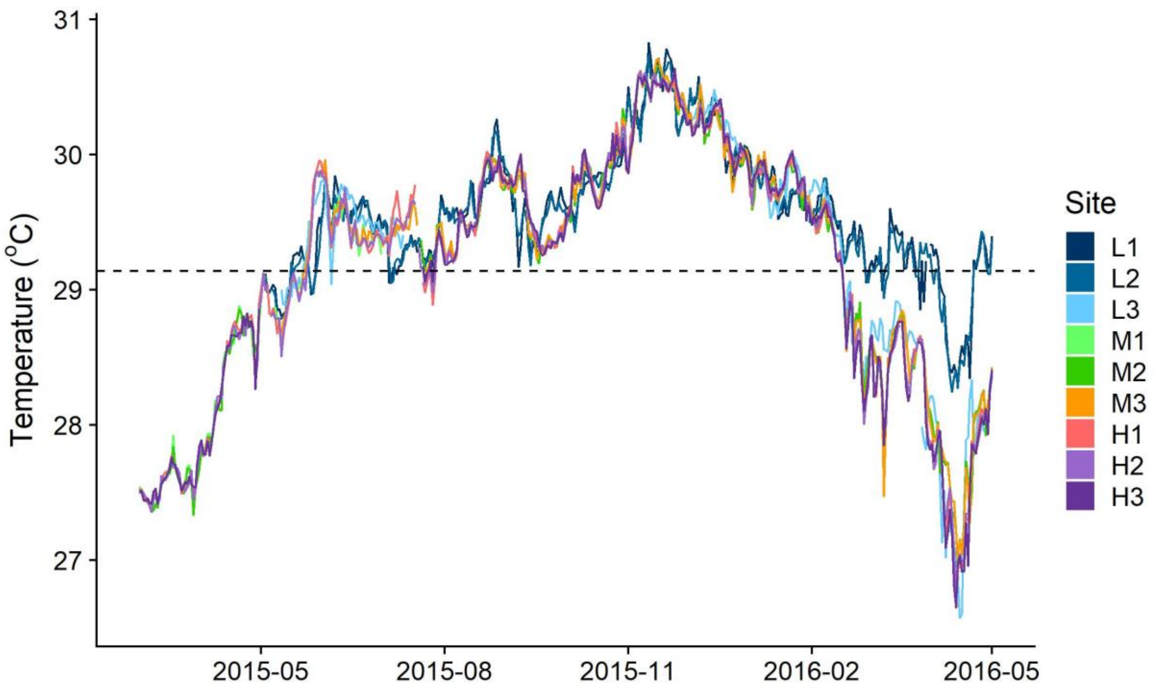

Figure S1. Mean daily water temperature (°C) at shallow forereef sites around Kiritimati throughout the 2015-2016 El Niño and mass coral bleaching event. Data were measured *in situ* using SBE-56 temperature loggers (Sea-Bird Electronics, Bellevue, USA). The dashed line represents 1°C above Kiritimati's historical maximum monthly temperature, indicative of the coral bleaching threshold.
